# Out of sight, out of mind: Understanding the sanitation crisis in global South cities

**DOI:** 10.1016/j.jenvman.2021.114285

**Published:** 2022-03-15

**Authors:** Victoria A. Beard, David Satterthwaite, Diana Mitlin, Jillian Du

**Affiliations:** a Cornell University, World Resources Institute, USA; b International Institute for Environmental Development, UK; c University of Manchester, International Institute for Environmental Development, UK; d World Resources Institute, USA

**Keywords:** Urban sanitation, Human waste, Infrastructure, Planning, Affordability, Public health

## Abstract

Global monitoring efforts do not provide a clear picture of the challenge of managing human waste at the city scale. Where cities do not provide universal access to publicly managed sanitation systems, households and communities find their own solutions resulting in a patchwork of approaches to removing human waste from places where people live. In dense urban environments, the absence of a coordinated approach can create serious public health problems. In the absence of comparable city-level data, we analyze primary and secondary data from 15 cities and 15 informal settlements in sub-Saharan Africa, South Asia, and Latin America. Across these regions, our study finds that 62 percent of human waste is not safely managed. We also find that, while many cities have a proportion of households connected to sewers, none of the 15 cities safely manage human waste at scale. In the absence of sewers, on-site fecal sludge management systems place enormous responsibility on households and private providers, and unaffordable sanitation options result in risky sanitation practices.

## Highlights

• In the cities studied 62 percent of human waste was not safely managed; none of the cities safely manage waste at scale.
• On-site sanitation places responsibility on households and private providers, and requires government capacity to regulate.
• In cities where sewers are not accessible, households engage in unsafe sanitation practices creating public health risks.

## 1 Introduction

This paper analyzes the urban sanitation service provision gap in cities in the global South. Sustainable Development Goal (SDG) 6 seeks to “ensure availability and sustainable management of water and sanitation for all,” and SDG target 6.2 aspires to “achieve access to adequate and equitable sanitation and hygiene for all and end open defecation by 2030” (UN DESA, 2017). Despite this goal, in 2017, in countries where sanitation data were available, global estimates report that only 47 percent of urban residents had access to safely managed sanitation (UNICEF and WHO, 2019). And, in most cities in the global South, sanitation infrastructure and services have not kept pace with population growth (Berendes et al., 2018; Mara and Evans, 2017).

The World Health Organization (WHO) 2018 guidelines underscore the link between access to safe sanitation and improvements in health and well-being (WHO, 2018). The health risks from unsafe sanitation practices are numerous and varied, and include infection and disease, stunting, and the emergence and spread of antimicrobial resistance (Bartram and Cairncross, 2010; WHO, 2018). An important consideration in densely populated urban areas is that improvements in household level sanitation facilities and practices have limited positive impacts on the overall health of the population if they fail to consider the potential for exposure along the entire sanitation chain (Berendes et al., 2020; Berendes et al., 2018; Mills et al., 2018; Robb et al., 2017; SuSanA, 2018).

The sanitation service chain starts with what happens to human waste where households reside. Where households do not have access to sewers to convey human waste away from the plot, the service chain begins with on-site containment and includes collection, transport, treatment plant, reuse, and disposal (Nelson and Murray, 2008). In many cities, untreated or partially treated human waste is at risk of “leaking” at various points along the sanitation service chain (Dasgupta et al., 2021; Devaraj et al., 2021; Mills et al., 2018).

From the household perspective there are well established, measurable health and economic benefits associated with access to safe sanitation (The Lancet, 2007). There is the reduced household expenditure on health care, such as the cost of treating diarrheal disease and other waterborne diseases from exposure to water contaminated with human waste (Berendes et al., 2018; Booth et al., 2018; Hutton, 2011). There is the benefit of time and earnings saved from lost productivity due to sanitation related illnesses as well as the time spent caring for sick family members (Perard, 2018). There is the economic benefit of savings related to reduced premature mortality (Perard, 2018). There is also the time saved when sanitation facilities are more accessible, including not having to wait for toilets (Booth et al., 2018; Hutton, 2011; WHO, 2012). Finally, there are savings for residents of informal settlements who often pay more to access lower quality sanitation facilities (Gaisie et al., 2018).

Sanitation provision in densely populated urban areas presents complex and unique challenges that are often ignored by researchers, policy makers, and municipalities (Dasgupta et al., 2021). In rapidly growing cities, urban informality in construction practices combined with the subterranean nature of on-site sanitation solutions, such as pits and septic tanks, means that households often lack a clear understanding of how these containment systems were constructed and maintained over time (Devaraj et al., 2021; Englund et al., 2020; Freihardt, 2020; Strande et al., 2018). Even where this information is known, households lack control over what happens to human waste after it leaves the home or plot (Berendes et al., 2018, 2020). Public health crises result when there are breakdowns in part of either formal or informal sanitation systems, and households have to “make-do” and resolve these problems on their own (Dirix et al., 2021; Yesaya and Tilley, 2021). In short, in contexts where households are left to find their own sanitation “solutions,” there is an increased risk of contamination to the population as a whole (Weststrate et al., 2019). Access to better quality sanitation will likely increase household expenditures either through sanitation tariffs (often included in the cost of water service), higher rental costs for accommodation, or as a charge to access public toilets, and urban policy makers have paid insufficient attention to the affordability aspect of sanitation acess.

There are significant environmental risks associated with the disposal or leakage of untreated human waste in densely populated urban areas. Leaks from underground sewers, ineffective septic tanks, and poorly constructed pit latrines can contaminate groundwater (Nyenje et al., 2013). Flooded pit latrines, open sewers, and open defecation all have the potential to contaminate surface water, which is strongly associated with the propagation of filariasis, a parasitic disease (Kimmelman, 2017; WHO, 2018). There is also the link between the release of untreated human waste into rivers, streams, and lakes and the rise of eutrophication, which is where natural water bodies become overly rich in plant biomass due to higher levels of nitrogen and phosphorus (Nyenje et al., 2010, 2013). Eutrophication is responsible for numerous negative outcomes, including water hyacinth blooms, the eradication of endemic fish species, and the rapid growth of phytoplankton blooms (Nyenje et al., 2010). In extreme cases this can result in monospecies blooms, like cyanobacteria, which adversely affects the health of humans and animals (Nyenje et al., 2010).

The article begins with a discussion of the limitations with global measurements most often used to assess progress on sanitation access and SDG 6.2. To address this gap, the article analyzes data from 15 cities and associated informal settlements in sub-Saharan Africa, South Asia, and Latin America. The article explains the methods used to collect and analyze the data. The discussion of the findings begins with a discussion of patterns in cities across three geographic regions. Then we describe the sanitation facilities that households use, how households manage their waste, and what happens to human waste. Finally, the analysis examines what households pay for sanitation and considers whether sanitation is affordable, particularly to low-income households. The article concludes with a summary of the main findings and a discussion of the relationship between the lack of universal access to publicly managed sanitation services and public health risk to all urban residents.

## 2 The urban sanitation crisis and why it is underestimated

SDG targets 6.1 and 6.2 focus on access to safely managed water and sanitation facilities. The World Health Organization and the United Nations Children's Fund, through the Joint Monitoring Programme (JMP), are mandated to monitor global progress on SDG 6. The JMP has created a service ladder that categorizes sanitation into two broad categories: improved or unimproved. Improved facilities are further subdivided between those that are shared between two or more households and those that are not shared. From the top of the “sanitation service ladder,” other categories include safely managed, followed by basic, limited, unimproved, and open defecation.

Unfortunately, these categories have a limited ability to inform sanitation policy and action in urban areas. The “safely managed” category includes pit latrines and septic tanks—sanitation practices which are extremely difficult to regulate and safely manage in densely populated settings with high poverty rates, informal construction, flooding, and limited municipal capacity for regulation, treatment, reuse, and disposal. And by not distinguishing between those approaches to sanitation that are appropriate in higher densities and those that are not appropriate, they fail to identify solutions that involve higher health risks in dense residential neighborhoods. Furthermore, the categories on the sanitation service ladder pay inadequate attention to the affordability of different sanitation options, so that households may have theoretical access to improved sanitation but may not be able to afford to use it. Finally, the categorization of all shared toilets as “limited” is problematic given the evidence on the contribution of well-constructed community-managed toilets in addressing the need for urban sanitation services (Bartram et al., 2018; Buckley and Kallergis, 2019; Jenkins et al., 2014).

Globally, data on the management and treatment of human waste have only recently been collected, and are only collected in a limited number of national contexts. Data from the Demographic and Health Survey (DHS) (on which much of the JMP statistics are based) and other national sample surveys have increased the scope and detail of questions asked about sanitation. However, data are still lacking regarding the spatial distribution of sanitation access in each urban area because most national surveys have sample sizes that are too small to provide relevant information at the city level. In addition, informal settlements are often ignored and thus underrepresented in surveys (Jenkins et al., 2014).

To fully understand access to urban sanitation and galvanize action, urban change agents need city and sub-city level data on sanitation practices and service provision at each step of the sanitation service chain. Persistent challenges to collecting sanitation data exist in urban areas where households do not have access to publicly managed sanitation systems, on-site sanitation practices are largely at the discretion of households (or landlords), and in cities where large parts of the urban fabric are constructed informally and the sanitation infrastructure is subterranean, and thus very difficult to assess the appropriateness and safety of its construction and maintenance (Devaraj et al., 2021). Neither the DHS nor the JMP currently provides this information.

In sum, three limitations make it difficult to accurately understand the risk that current sanitation practices pose to urban populations:

1. The UN category of “improved” sanitation encompasses such a wide variety of sanitation practices that it does not provide a useful picture of the public health risks in urban areas.
2. The JMP categories fail to address affordability from the perspective of the household, especially low-income households.
3. Most cities have missing data about sanitation practices and management at different stages of the service chain, and even less information available about sanitation practices in informal settlements.

In the absence of comparable city-level sanitation data, we collected data in 15 cities and 15 corresponding informal settlements in the global South.^1^

## 3 Research design, methods, and data

To obtain a picture of city-level sanitation, we collected and analyzed existing secondary data and collected new data from 15 cities located in sub-Saharan Africa, South Asia, and Latin America. The 15 cities are Kampala, Uganda; Lagos, Nigeria; Maputo, Mozambique; Mzuzu, Malawi; Nairobi, Kenya; Bengaluru, India; Colombo, Sri Lanka; Dhaka, Bangladesh; Karachi, Pakistan; Mumbai, India; Caracas, Venezuela; Cochabamba, Bolivia; Rio de Janeiro, Brazil; São Paulo, Brazil; and Santiago de Cali, Colombia. The cities were selected to represent the three major regions of the global South and the diversity of demographic profiles, patterns of urbanization, and levels of economic development achieved in each of these three regions. This study was done as part of a larger research project for the World Resources Institute, *Towards a More Equal City* (Beard et al., 2016).

The data in Table 1, Table 2, Table 3, Table 4, Table 5 are based on research we did in collaboration with local researchers in each city. The data in the tables are combined from publicly available data sets, administrative records, websites, and project documents. In addition, researchers in each city conducted an average of seven key informant interviews to fill in gaps where publicly available data were missing. At the city level, data were compiled about household sanitation practices and access to facilities, citywide sanitation infrastructure, the cost of on-site sanitation construction, fecal sludge removal, fees for accessing piped sewers, and the construction of on-site sanitation systems and proximity to water sources.

**Table 1:** Population characteristics in 15 cities and informal settlements in the global South.^b^.

City name	Country	Type of jurisdiction	Population	Average household size	Urban density (people/sq. km)	% of workforce in informal economy	% of house-holds in informal settlement	Avg house-hold income/month (USD)	Informal settlement name	Popu-lation	Avg. household size	Urban density (people/sq. km)	Average house-hold income/month ($USD)
Kampala	Uganda	city	1,507,080	4.0	7974	70	60	124	Kalimali	1540	5.0	48	48
Lagos	Nigeria	metropolis	23,300,000	5.0	19,898	70	70	218	Makoko	204,720	5.0	96,566	195
Maputo	Mozambique	municipality	1,194,121	4.9	3431	55	9	162	Nhlamankulu D	12,175	5.1	2202	130
Mzuzu	Malawi	city	254,891	5.0	1770	80	60	91	Zolozolo West Ward	21,349	5.0	10,215	81
Nairobi	Kenya	city county	4,397,073	3.2	6421	53	65	213	Kosovo Village in Mathare Valley	12,000	3.0	120,000	81
Bengaluru	India	municipality	8,443,675	4.0	11,395	60	30	668	Koramangala Slum (Resettlement) Cluster	38,500	4.5	140,000	179
Colombo	Sri Lanka	municipality	555,031	6.1	15,001	38	44	549	Borella South	5127	4.2	754	503
Dhaka	Bangladesh	city corporation	6,970,105	4.4	22,778	75	23	653	Kallyanpur Pora Basti	11,357	3.9	227,140	171
Karachi	Pakistan	municipality	16,054,988	6.0	12,350	70	52	330	Ghaziabad Sector 11 ½, Orangi Town	51,000	8.0	78,462	273
Mumbai	India	municipal corporation	12,442,373	4.5	27,167	80	40	244	Siddarth Nagar	2160	4.2	–	202
Caracas^a^	Venezuela	municipality	3,319,849	3.7	4216	28	60	1803	Terrazas del Alba	3500	3.5	35,000	1075
Cochabamba	Bolivia	municipality	632,013	3.0	1612	55	27	210	San Miguel Km4	1705	6.0	131	168
Rio de Janeiro	Brazil	municipality	6,320,446	3.0	5263	35	23	475	Rocinha	77,178	3.0	90,798	378
São Paulo	Brazil	municipality	12,040,000	3.2	7916	20	12	1083	Jardim São Remo	6930	3.5	86,500	410
Santiago de Cali	Colombia	municipality	2,278,022	4.0	3680	60	23	437	Comuna 20	68,980	4.0	n/a	195

Note: n/a = not applicable. Figures for population, households, and average household size are based on national statistics. Figures for percentage of workforce in informal economy, households in informal settlements, and average household incomes were locally determined. These figures came from a combination of key informants, project reports, and government records. Informal settlements were selected based on the following criteria: 1) centrally located within the city, and 2) represents an average or typical informal settlement in the city.

a Caracas has variable inflation rates. Costs were converted using the exchange rate at data collection: 2012 (Bs 4.30 to US$1).

b **Caracas:** INE (Instituto Nacional de Estadística) - INE - Venezuela, 2011a. Censo 2011. http://www.ine.gob.ve/index.php?option=com_content&view=category&id=95&Itemid=26. Interview: Community leader, Terrazas del Alba, July 2017. **Cochabamba:** INE (Instituto Nacional de Estadística) - INE - Bolivia, 2011. ENCUESTA ECONÓMICA ANUAL Industria Manufacturera, Comercio y Servicios EECON 2011. Instituto Nacional de Estadística, La Paz. Interview: Administrator, Drinking Water Association of San Miguel Km4, March 2017. **Rio de Janeiro:**IBGE (Instituto Brasileiro de Geografia e Estatística), 2010. Census 2010 (Online Database). http://censo2010.ibge.gov.br/. IBGE, 2009. Final report of the household census of the Rocinha Complex Rio de Janeiro - Dec/2009 PAC Rocinha. IBGE, Rio de Janeiro.; Interviews: Community leaders, Rocinha, July 2017. **São Paulo:**CEM (Centro de Estudos da Metrópole), n.d. http://www.fflch.usp.br/centrodametropole/1289, IBGE (Instituto Brasileiro de Geografia e Estatística), 2010. Census 2010 (Online Database). http://censo2010.ibge.gov.br/, IBGE (Instituto Brasileiro de Geografia e Estatística), 2010. GeoSampa/ Prefeitura Municipal de São Paulo, 2017. Mapa Digital da Cidade de São Paulo (Online Database). Prefeitura Municipal de São Paulo. http://geosampa.prefeitura.sp.gov.br/PaginasPublicas/_SBC.aspx#, [Bibr bib60][Bibr bib58]. Map of São Paulo (Online Database). Prefeitura Municipal de São Paulo. http://mapa.habitasampa.inf.br/, IBGE (Instituto Brasileiro de Geografia e Estatística), 2010. Census 2010 (Online Database). http://censo2010.ibge.gov.br/. **Santiago de Cali:**Municipality of Santiago de Cali, 2017. Cali en Cifras (Online Database). http://www.cali.gov.co/planeacion/publicaciones/137802/cali-en-cifras/. **Bengaluru:**Ministry of Home Affairs, n.d. 2011 Census data. Government of India. http://censusindia.gov.in/2011-Common/CensusData2011.html.; Interview: Community leader, Slum Jagatthu, July 2017; Community leader, Koramangala, July 2017. **Mumbai:**Directorate of Census Operations, 2011. District Census Handbook: Mumbai. Series-28. Census of India, Mumbai., WIEGO, 2018. http://www.wiego.org/dashboard/statistics/south-asia/india/mumbai. ICDS (Integrated Child Development Services Scheme), 2016. Siddarth Nagar Slum Survey, Mumbai. **Colombo:**Department of Census and Statistics, 2017. Census Of Population and Housing 2011. Government of Sri Lanka. http://www.statistics.gov.lk/PopHouSat/CPH2011/index.php. **Karachi:**Pakistan Bureau of Statisitcs, 2017. Population Census 2017. Government of Pakistan. http://www.pbscensus.gov.pk/. Interviews: Representative, Technical Training Resource Centre; Community leaders, Ghaziabad Sector, July 2017. **Dhaka:**Bangladesh Bureau of Statistics, 2011. Population and Housing Census. WSUP, 2016a. Dhaka Survey - Kallyanpur. WSUP. **Kampala:**Government of Uganda, 2016. national population and housing census 2014, Main report. UBOS (Uganda Bureau of Statistics), Kampala. ACTogether Uganda, NSDFU (National Slum Dwellers Federation of Uganda), SDI (Slum Dwellers International), n.d. Kampala Profiles, Kawempe Division. http://www.actogetherug.org/. **Lagos:**LBS (Lagos Bureau of Statistics), 2013. Household Survey 2013 Main Report. Ministry of Economic Planning and Budget, Lagos State Government. **Maputo:** Population figure is based on preliminary results of the 2017 census; Conselho Municipal de Maputo (2010). Perfil Estatístico do Município de Maputo, Maputo., INE (Instituto Nacional de Estatística) - INE - Mozambique, 2012. Inquérito Contínuo aos Agregados Familiares, Maputo., INE (Instituto Nacional de Estatística) - INE - Mozambique, 2006. Primeiro Inquérito Nacional ao Sector Informal, Maputo., INE (Instituto Nacional de Estatística) - INE - Mozambique, 2010. Perfil Estatístico Do Município 2007–2008 Maputo, Maputo. Conselho Municipal de Maputo, 2010. Perfil Estatístico do Município de Maputo, Maputo., INE (Instituto Nacional de Estatística) - INE - Mozambique, 2015. Relatório Final do Inquérito ao Orçamento Familiar - IOF-2014/15., WSUP, 2016b. Relatório de Água e Saneamento dos 11 Bairros do Distrito de Nhalhamankulu pelos Líderes Locais – II Levantamento, Maputo. **Mzuzu:**Mzuzu City Council, 2017. Environmental Report, Administrative records, Mzuzu., National Statistical Office, 2010. Population and Housing Census 2008, Spatial Distribution and Urbanisation. National Statistical Office, Zomba., UN Habitat, 2011. Malawi: MZUZU Urban PrOFilE. **Nairobi:**KNBS (Kenya National Bureau of Statistics), 2010. The 2009 Kenya Population and Housing Census: Vol II: Population and Household Distribution by Socio-Economic Characteristics. KNBS, Nairobi., KNBS (Kenya National Bureau of Statistics), 2018. Statistical Abstract 2017. KNBS, Nairobi., Nairobi City County et al., 2014. Nairobi Integrated Urban Development Master Plan. Nairobi., World Bank, 2016. Kenya Urbanization Review: Nairobi Statistical Abstract. The World Bank, Nairobi. http://documents.worldbank.org/curated/en/639231468043512906/pdf/AUS8099-WP-P148360-PUBLIC-KE-Urbanization-ACS.pdf.Interview: Community leader, Kosovo Village, July 2017.

**Table 2:** Household access to urban sanitation facilities.^b^.

City name	City-wide: percentage of households that use […]				Informal settlement name	Selected informal settlements: percentage of households that use […]			
	Private	Shared	Communal/public	No facilities/other		Private	Shared	Communal/public	No facilities/other
Kampala	30.0	60.0	10.0	0.0	Kalimali	5.0	5.0	90.0	0.0
Lagos	27.0	72.0	0.0	1.0	Makoko	20.0	65.0	0.0	15.0
Maputo	90.0	10.0	0.0	0.0	Nhlamankulu D	79.0	15.0	5.0	1.0
Mzuzu^a^	47.0	51.0	0.0	2.0	Zolozolo West Ward	60.0	30.0	0.0	10.0
Nairobi	27.0	45.0	28.0	0.0	Kosovo Village in Mathare Valley	5.0	85.0	10.0	0.0
Bengaluru	97.0	0.0	1.5	1.5	Koramangala	86.0	0.0	13.0	1.0
Colombo	80.0	4.0	16.0	0.0	Borella South	93.0	1.5	5.5	0.0
Dhaka	73.0	22.0	5.0	0.0	Kallyanpur Pora Basti	0.0	27.0	73.0	0.0
Karachi	90.0	3.0	0.0	7.0	Ghaziabad Sector 11½, Orangi Town	100.0	0.0	0.0	0.0
Mumbai	56.0	0.0	33.0	11.0	Siddarth Nagar	15.0	30.0	0.0	55.0
Caracas	99.5	0.0	0.0	0.5	Terrazas del Alba	100.0	0.0	0.0	0.0
Cochabamba	100.0	0.0	0.0	0.0	San Miguel Km4	100.0	0.0	0.0	0.0
Rio de Janeiro	95.0	2.0	2.5	0.5	Rocinha	97.0	1.0	0.0	2.0
São Paulo	100.0	0.0	0.0	0.0	Jardim São Remo	100.0	0.0	0.0	0.0
Santiago de Cali	99.0	0.0	1.0	0.0	Comuna 20	99.0	0.0	1.0	0.0

Note: Private sanitation facility is defined as a facility is in household/dwelling. Shared sanitation facility is defined as privately managed and shared between more than one household. Communal/public sanitation facility is defined as community-managed or government-managed facility. No facilities/open is defined as no facilities, open defecation.

a Based on key informant estimates derived from a study limited to three communities.

b **Caracas:** INE (Instituto Nacional de Estadística) - INE - Venezuela, 2011b. Informe Geoambiental 2011 Distrito Capital. http://www.ine.gob.ve/index.php?option=com_content&view=category&id=68&Itemid=49#.; Interviews: Community leaders, Terrazas del Alba, July 2017. **Cochabamba:** Interview: Regulator, National Water Authority; Administrator, Drinking Water Association, San Miguel Km4, March 2017. **Rio de Janeiro:** Interview: Manager, CEDAE - STS Alegria; Interviews: Director, Rocinha Health Office, July 2017. **São Paulo:**IBGE, 2010. São Paulo (Online Database). IGBE. https://cidades.ibge.gov.br/brasil/sp/sao-paulo/panorama. **Santiago de Cali:**Municipality of Santiago de Cali, 2017. Cali en Cifras (Online Database). http://www.cali.gov.co/planeacion/publicaciones/137802/cali-en-cifras/. Interview: Community leader, Comuna 20, July 2017. **Bengaluru:**BWSSB (Bangalore Water Supply and Sewerage Board), 2011. National Census 2011 – Household Amenities table 14. BWSSB.; Interviews: Community leader, Koramangala, July 2017. **Mumbai:** Interview: Officer, Sanitation Department, MCGM; Community leaders, Siddarth Nagar; July 2017. **Colombo:** Interview: Engineer, Colombo Municipal Council; Administrator, Grama Niladhari, Borella South, July 2017; Department of Census and Statistics, 2017. Census Of Population and Housing 2011. Government of Sri Lanka. http://www.statistics.gov.lk/PopHouSat/CPH2011/index.php. **Karachi:** Interview: Director, Orangi Pilot Project; Community leaders, Ghaziabad, July 2017; **Dhaka:** Interviews: Representatives, DWASA; Consultant, World Bank, Sep 2017; Bangladesh Bureau of Statistics, 2015. Census of Slum Areas and Floating Population. **Kampala:** Interview: Representatives, KCCA and ACTogether; Community leaders, Kalimali, July 2017. **Lagos:**Lagos Bureau of Statistics, 2016. Household Survey. Lagos Bureau of Statistics.; Interview: Makoko Representative, Nigeria Red Cross Society, July 2017. **Maputo:** Interview: Water and Sanitation Specialist, World Bank; Sanitation manager, Municipality Drainage Office, July 2017; Water and Sanitation Program Municipality of Maputo, 2014. Caracterização do Saneamento em Maputo, Maputo. **Mzuzu:**Manda, 2009. Water and Sanitation in Urban Malawi- Can the Millennium Development Goals be Achieved? A study of Informal Settlements in three cities. IIED, London.; Interviews: Community leaders, Zolozolo West Ward, July 2017. **Nairobi:** Interviews: Community leaders, Kosovo Village, May 2017; World Bank (2016). Kenya Urbanization Review: Nairobi Statistical Abstract. The World Bank, Nairobi. http://documents.worldbank.org/curated/en/639231468043512906/pdf/AUS8099-WP-P148360-PUBLIC-KE-Urbanization-ACS.pdf.

**Table 3:** Household access to urban sanitation infrastructure and services.

City name	Piped water availa-bility	Percentage of households that use […]^e^							Informal settlement name	Piped water availa-bility	Percentage of households that use […]^f^						
	hours per day/days per week	Se-wer	Pri-vate sep-tic tank	Com-munal septic tank	Pit la-trine	Self-provi-sioned drain to water-way	Compos-ting, bucket, hanging toilet	Open defeca-tion		hours per day/days per week	Se-wer	Pri-vate sep-tic tank	Com-munal septic tank	Pit la-trine	Self-provi-sioned drain to water-way^a^	Compos-ting, bucket, hanging toilet	Open defeca-tion
Kampala	8/7	10	20	5	60	–	5	0	Kalimali	0/0	0	20	0	80	0	0	0
Lagos	varies	0	61	0	34	2	1	2	Makoko	0/0	0	25	0	75	0	0	0
Maputo	10/7	9	49	0	41	0	0	1	Nhlamankulu D	6/7	0	44	0	56	0	0	0
Mzuzu	20/7	0	16	0	84	0	0	0	Zolozolo West Ward	20/7	0	5	0	95	0	0	0
Nairobi	18/5	48	8	3	41	–	0	0	Kosovo Village in Mathare Valley	24/7	0	0	0	1	99	0	0
Bengaluru	3/3	79	6	0	11	–	1	3	Koramangala	2/2.5	100	0	0	0	0	0	0
Colombo	24/7	39	59	0	1	–	0	1	Borella South	24/7	51	49	0	0	0	0	0
Dhaka^d^	24/7	18	75^c^	3	4	0	0	0	Kallyanpur Pora Basti	24/7	0	12^c^	85	3	0	0	0
Karachi	2/3	75	6	4	0	–	0	15	Ghaziabad Sector 11 ½, Orangi Town	1/1.5	0	0	0	0	100	0	0
Mumbai	7/7	28	28	20	1	13	0	10	Siddarth Nagar	0/0	0	0	0	0	45	0	55
Caracas	13/7	97	2	0	0	–	0	1	Terrazas del Alba	4/3	100	0	0	0	0	0	0
Cochabamba	15/3	80	20	0	0	–	0	0	San Miguel Km4	4/4	90	10	0	0	0	0	0
Rio de Janeiro	20/7	53	16	0	1	25	0	5	Rocinha	12/4	0	1	0	2	91^b^	0	6
São Paulo	24/7	87	2	0	0	11	0	0	Jardim São Remo	24/7	40	1	0	0	59	0	0
Santiago de Cali	24/7	99	0	0	1	–	0	0	Comuna 20	24/7	99	0	0	1	0	0	0

a Estimates of fecal sludge disposal using “self-provisioned drain to nearby waterway” are more accurate for the informal settlements because enumerators conducted field research and direct observation in these settlements.

b In dry weather, sewage is combined with drainage and goes to a river emissary; in rainy weather, it is drained directly into the sea.

c More than 80% are septic tanks without soak pits and directly connected to storm water drainage.

d In Dhaka, although pressure is intermittent, water is available at all times, but residents use hand or electric pump to obtain water from the piped network.

e **Caracas:** Interview: Former advisor, Hidrocapital, July 2017; INE (Instituto Nacional de Estadística) - INE - Venezuela, 2011a. Censo 2011. http://www.ine.gob.ve/index.php?option=com_content&view=category&id=95&Itemid=26. **Cochabamba:** Interview: Environmental Regulator and Water Engineer, National Water Authority, March 2017. **Rio de Janeiro:** Interview: Manager, CEDAE - STS Alegria; Manager, Rio Aguas Foundation; Director, CEDAE, July 2017; IBGE (Instituto Brasileiro de Geografia e Estatística), 2010. Census 2010 (Online Database). http://censo2010.ibge.gov.br/. **São Paulo:** Interview: Specialist in São Paulo household construction, July 2017; ANA (National Water Agency), 2017. Atlas Esgotos (Online Database). ANA. http://www.snirh.gov.br/portal/snirh/snirh-1/atlas-esgotos. **Santiago de Cali:**Emcali (2017). Emcali. www.emcali.com, Municipality of Santiago de Cali, 2018. http://www.cali.gov.co/dagma/. **Bengaluru:**BWSSB (Bangalore Water Supply and Sewerage Board), 2011. National Census 2011 – Household Amenities table 14. BWSSB. **Mumbai:** Interview: Officer, Sanitation Department, MCGM; Professor, Center of Water Policy, Regulation and Governance, Tata Institute of Social Sciences, July 2017; Directorate of Census Operations, 2011. District Census Handbook: Mumbai. Series-28. Census of India, Mumbai. **Colombo:** Interview: Engineer, Colombo Municipal Council, July 2017; Department of Census and Statistics, 2017. Census Of Population and Housing 2011. Government of Sri Lanka. http://www.statistics.gov.lk/PopHouSat/CPH2011/index.php. **Karachi:** Interview: Staff representative, Orangi Pilot Project; Community leaders of Orangi and Baldia; Director, Urban Resource Center; Senior Engineers at Karachi Water and Sewerage Board (KWSB), July 2017; **Dhaka:** Interviews: Consultant, World Bank; Representative, WSUP-Dhaka, July 2017; WSUP, 2015. Financial Analysis and Business Model Development on FSM Service. WSUP and UNICEF., WSUP-World Bank, 2014. Base line Survey: Bangladesh LIC WASH Programme, 2014. World Bank. **Kampala:** Interview: Representatives, NWSC; Representatives, KCCA; Representative, ACTogether; Representative, CIDI, July 2017. **Lagos:** Interview: Manager, Lagos State Waste Water Office, July 2017; LBS (Lagos Bureau of Statistics), 2013. Household Survey 2013 Main Report. Ministry of Economic Planning and Budget, Lagos State Government. **Maputo:** Interview: Water and Sanitation Specialist, World Bank; Sanitation Manager, Municipality Drainage Office; Planning Technical Officer, Águas da Região de Maputo, July 2017; Water and Sanitation Program Municipality of Maputo, 2014. Caracterização do Saneamento em Maputo, Maputo. **Mzuzu:** Interview: Officer, National Statistics Office; Representative, City Council July 2017; National Statistical Office (2010). Population and Housing Census 2008, Spatial Distribution and Urbanisation. National Statistical Office, Zomba. **Nairobi:**KNBS (Kenya National Bureau of Statistics), 2012. 2009 Kenya Population and Housing Census. Analytical Report on Urbanization. KNBS, Nairobi., Majidata (2017). Water and sanitation data portal. http://majidata.go.ke/wsb.php?MID=MTE=&SMID=NQ==.

f **Caracas:** INE (Instituto Nacional de Estadística) - INE - Venezuela, 2011b. Informe Geoambiental 2011 Distrito Capital. http://www.ine.gob.ve/index.php?option=com_content&view=category&id=68&Itemid=49#.; Interviews: Community leaders, Terrazas del Alba, July 2017. **Cochabamba:** Interview: Regulator, National Water Authority; Administrator, Drinking Water Association, San Miguel Km4, March 2017. **Rio de Janeiro:** Interview: Manager, CEDAE - STS Alegria; Interviews: Director, Rocinha Health Office, July 2017. **São Paulo:**[Bibr bib60][Bibr bib58]. Map of São Paulo (Online Database). Prefeitura Municipal de São Paulo. http://mapa.habitasampa.inf.br/, IBGE, 2010. São Paulo (Online Database). IGBE. https://cidades.ibge.gov.br/brasil/sp/sao-paulo/panorama, Sabesp (2018). Sabesp. http://site.sabesp.com.br/site/Default.aspx. **Santiago de Cali:**Municipality of Santiago de Cali, 2017. Cali en Cifras (Online Database). http://www.cali.gov.co/planeacion/publicaciones/137802/cali-en-cifras/. Interview: Community leader, Comuna 20, July 2017. **Bengaluru:**BWSSB (Bangalore Water Supply and Sewerage Board), 2011. National Census 2011 – Household Amenities table 14. BWSSB.; Interviews: Community leader, Koramangala; Representative, BWSSB, July 2017. **Mumbai:** Interviews: Community leaders, Siddarth Nagar; Campaign leaders, Pani Haq Samiti (Committee for Water Right), July 2017. **Colombo:** Interview: Statistician, Department of Census and Statistics; Administrator, Grama Niladhari, Borella South, July 2017; Department of Census and Statistics, 2017. Census Of Population and Housing 2011. Government of Sri Lanka. http://www.statistics.gov.lk/PopHouSat/CPH2011/index.php. **Karachi:** Interviews: Community leaders, Ghaziabad, July 2017. **Dhaka:** Interviews: Community leaders, Kallyanpur Pora Basti; Representative, NDBUS (Slum Committees), Sep 2017; PMID (Participatory Management Initiative for Development), 2013. Analysis of factors affecting the usage, operation and maintenance of community latrines in low income communities (lics), in Dhaka city, for WSUP Bangladesh LIC WASH Programme 2013–15. **Kampala:** Interview: Representatives, KCCA and ACTogether; Community leaders, Kalimali, July 2017. **Lagos:** Interview: Makoko Representative, Nigeria Red Cross Society; Representative, Lagos State Waste Water Office, July 2017; LBS (Lagos Bureau of Statistics), 2013. Household Survey 2013 Main Report. Ministry of Economic Planning and Budget, Lagos State Government. **Maputo:** Interview: Water and Sanitation Specialist, World Bank; Sanitation manager, Municipality Drainage Office, July 2017. **Mzuzu:** Interviews: Community leaders, Zolozolo West Ward, July 2017. **Nairobi:** Interviews: Community leaders, Kosovo Village, May 2017.

**Table 4:** Cost of on-site sanitation construction and fecal sludge removal.

City name^f^	Method	Construc-tion costs ($USD)	Removal method	Cost to empty one time ($USD)	Informal settlement name^g^	Method	Construc-tion costs ($USD)	Removal method	Cost to empty one time ($USD)
Kampala	Private septic tank 4-stance^a^	3299	Pump-out	58	Kalimali	Private septic tank	687	Pump-out	76
	Communal septic tank 4-stance	4123	Pump-out	58					
	Pit latrine 4-stance VIP 4-stance slab	1457 1405	Pump-out, small gulper tech, no removal	10; 0		Pit latrine	687	Pump-out; no removal	96; 0
	Composting toilet	2749	Manual	10					
Lagos	Private septic tank	1490	Pump-out	53	Makoko	Private septic tank	1490	pump-out	45
	Pit latrine VIP slab no slab	1192 89 75	Manual: VIP slab Buried, no removal	45 16 0		Pit latrine VIP slab	1192 894	pump-out	45
	Hanging latrine	0	Direct to waterway	0					
Maputo	Private septic tank	462	Small gulper tech Manual Pump-out (vacuum truck)	23 8 97	Nhlamankulu D	Private septic tank	118	Small gulper tech Manual	23 8
	Pit latrine slab no slab	70 16	Small gulper tech Manual	23 8		Pit latrine slab no slab	70 16	Small gulper tech Manual	23 8
Mzuzu	Private septic tank	683	Pump-out	25	Zolozolo West Ward	Private septic tank	615	Pump-out	25
	Pit latrine VIP slab no slab	410 205 68	Flooding out; no removal	27; 0		Pit latrine VIP Slab no slab	410 137 68	No removal	0
	Composting toilet	273	Manual, no removal	0		Composting toilet	273	Manual, no removal	0
Nairobi	Private septic tank concrete plastic bio-digester	1987 662	Pump-out	43	Kosovo Village in Mathare Valley	Pit latrine	Slab: 189	Manual	7
	Communal septic tank	2838	Pump-out	43		Self-provisioned drain	114	Directly to waterway	Shack: 118 Block: 213
	Pit latrine VIP slab	402 378	Manual, pump-out, small gulper tech; flooding out	43; 0					
Bengaluru	Private septic tank	427	Pump-out	29	Koramangala	No on-site methods			
	Pit latrine	89	Pump-out	19					
Colombo	Private septic tank	645	Manual, pump-out	24	Borella South	Private septic tank	968	Pump-out	23
	Pit latrine	24	Manual, pump-out	24					
Dhaka	Private septic tank	593	Manual, connected to drainage	24	Kallyanpur Pora Basti	Private septic tank	593	Manual, pump-out, connected to drainage	24
	Communal septic tank	711	Manual Vacutug truck Connected to drainage	59 95 0		Communal septic tank	2135	Manual, pump-out	96
	Pit latrine Slab single pit	24	Manual	18		Pit latrine	36	Manual	6
Karachi	Private septic tank^b^	165	Manual	565	Ghaziabad Sector 11 ½, Orangi Town	Self-provisioned drain	122	direct to waterway	28
	Communal septic tank	108	Manual	47					
Mumbai	Private septic tank	466	Manual	116	Siddarth Nagar	Self-provisioned drain	23	Direct to waterway	0
	Communal septic tank	349	No removal	0					
	Pit latrine VIP no slab	70 23	No removal	0					
	Self-provisioned drain	171	Direct to waterway	0					
Caracas^c^	Private septic tank	485	Pump out, manual	97	Terrazas del Alba	No on-site methods			
Cochabamba	Private septic tank	210	Pump out, manual	97	San Miguel Km4	Private septic tank	280	Manual	210
Rio de Janeiro	Private septic tank	733	Pump-out	141	Rocinha	Private septic tank^e^	n/a	Pump-out	141
	Pit latrine No slab (10 seats)	879	No removal	0		Pit latrine	n/a	No removal	0
	Self-provisioned drain	612	Direct to waterway	0		Self-provisioned drain	532	Direct to waterway	0
São Paulo	Private septic tank	625	Pump-out, manual, gulper tech	313	Jardim São Remo	Private septic tank	312	Vacuum truck	219
	Self-provisioned drain	281	direct to waterway	0		Self-provisioned drain	156	Direct to waterway	0
Santiago de Cali	pit latrine^d^	–	no removal	0	Comuna 20	pit latrine^d^	–	no removal	0

Note: n/a = not applicable; VIP = ventilated improved pit. All costs reported in U.S. dollars. Currency figures were converted to U.S. dollars using market exchange rates corresponding to the time of data collection.

a 4-stance toilet blocks are typically shared latrines for rentals. Based on the selected informal settlement, 4-stance toilet blocks serve 8 to 15 households. Individual households do not pay for the cost of construction. In some areas of the city, households will construct 2-stance toilet blocks but an average cost of 2-stance toilets was not available.

b Private septic tank costs are higher than communal septic tanks because septic tanks are typically constructed on land that is publicly owned or with no administrative authorization for construction, and the private owner must pay an extra amount to the municipal official to earn permission. Some communities work collectively and are able to negotiate a better deal with the municipal staff.

c Costs from Caracas were converted using the black-market exchange rate during the time of data collection: Dec 2017 (Bs103,024 to 1 USD).

d Pit latrines in Cali are now abolished; construction costs are not available.

e Private septic tanks are no longer constructed. For the few buildings with private septic tanks, the cost was included in the building cost.

f **Caracas:** INE (Instituto Nacional de Estadística) - INE - Venezuela, 2011a. Censo 2011. http://www.ine.gob.ve/index.php?option=com_content&view=category&id=95&Itemid=26. **Cochabamba:** Interview: Environmental Regulator, National Water Authority, March 2017. **Rio de Janeiro:** Interview: Manager, CEDAE - STS Alegria; Manager, Rio Aguas Foundation, July 2017; IBGE (Instituto Brasileiro de Geografia e Estatística), 2010. Census 2010 (Online Database). http://censo2010.ibge.gov.br/. **São Paulo:** Interview: Specialist in São Paulo household construction, Sep 2018. **Santiago de Cali:**Emcali (2017). Emcali. www.emcali.com. **Bengaluru:**BWSSB (Bangalore Water Supply and Sewerage Board), 2011. National Census 2011 – Household Amenities table 14. BWSSB. **Mumbai:** Interview: Officer, Sanitation Department, MCGM; Directorate of Census Operations, 2011. District Census Handbook: Mumbai. Series-28. Census of India, Mumbai. **Colombo:** Interview: Engineer, Colombo Municipal Council. July 2017. **Karachi:** Interview: Staff representative, Orangi Pilot Project; Community leaders of Orangi and Baldia; Director, Urban Resource Center, July 2017; **Dhaka:** Interviews: Consultant, World Bank; Representative, WSUP-Dhaka, July 2017; WSUP, 2015. Financial Analysis and Business Model Development on FSM Service. WSUP and UNICEF., WSUP-World Bank, 2014. Base line Survey: Bangladesh LIC WASH Programme, 2014. World Bank. **Kampala:** Interview: Representatives, NWSC; Representatives, KCCA; Representative, ACTogether; Representative, CIDI, July 2017. **Lagos:** Interview: Manager, Lagos State Waste Water Office, July 2017; LBS (Lagos Bureau of Statistics), 2013. Household Survey 2013 Main Report. Ministry of Economic Planning and Budget, Lagos State Government. **Maputo:** Interview: Water and Sanitation Specialist, World Bank; Sanitation Manager, Municipality Drainage Office, July 2017; Water and Sanitation Program Municipality of Maputo, 2014. Caracterização do Saneamento em Maputo, Maputo. **Mzuzu:** Interview: Officer, National Statistics Office, July 2017; National Statistical Office (2010). Population and Housing Census 2008, Spatial Distribution and Urbanisation. National Statistical Office, Zomba. **Nairobi:**KNBS (Kenya National Bureau of Statistics), 2012. 2009 Kenya Population and Housing Census. Analytical Report on Urbanization. KNBS, Nairobi.

g **Caracas:** INE (Instituto Nacional de Estadística) - INE - Venezuela, 2011b. Informe Geoambiental 2011 Distrito Capital. http://www.ine.gob.ve/index.php?option=com_content&view=category&id=68&Itemid=49#.; Interviews: Community leaders, Terrazas del Alba, July 2017. **Cochabamba::** Interview: Regulator, National Water Authority; Administrator, Drinking Water Association, San Miguel Km4, March 2017. **Rio de Janeiro:** Interview: Manager, CEDAE - STS Alegria; Interviews: Director, Rocinha Health Office, July 2017. **São Paulo:**IBGE, 2010. São Paulo (Online Database). IGBE. https://cidades.ibge.gov.br/brasil/sp/sao-paulo/panorama. **Santiago de Cali:**Municipality of Santiago de Cali, 2017. Cali en Cifras (Online Database). http://www.cali.gov.co/planeacion/publicaciones/137802/cali-en-cifras/. Interview: Community leader, Comuna 20, July 2017. **Bengaluru:**BWSSB (Bangalore Water Supply and Sewerage Board), 2011. National Census 2011 – Household Amenities table 14. BWSSB.; Interviews: Community leader, Koramangala, July 2017. **Mumbai:** Interviews: Community leaders, Siddarth Nagar; July 2017.**Colombo:**Department of Census and Statistics, 2017. Census Of Population and Housing 2011. Government of Sri Lanka. http://www.statistics.gov.lk/PopHouSat/CPH2011/index.php. **Karachi:** Interviews: Community leaders, Ghaziabad, July 2017. **Dhaka:** Interviews: Community leaders, Kallyanpur Pora Basti; Representative, NDBUS (Slum Committees), Sep 2017; PMID (Participatory Management Initiative for Development), 2013. Analysis of factors affecting the usage, operation and maintenance of community latrines in low income communities (lics), in Dhaka city, for WSUP Bangladesh LIC WASH Programme 2013–15. **Kampala:** Interview: Representatives, KCCA and ACTogether; Community leaders, Kalimali, July 2017. **Lagos:** Interview: Makoko Representative, Nigeria Red Cross Society; Representative, Lagos State Waste Water Office, July 2017. **Maputo:** Interview: Water and Sanitation Specialist, World Bank; Sanitation manager, Municipality Drainage Office, July 2017. **Mzuzu:** Interviews: Community leaders, Zolozolo West Ward, July 2017. **Nairobi:** Interviews: Community leaders, Kosovo Village, May 2017.

**Table 5:** Cost of construction and connection fees of sanitation facilities connected to piped sewers.

City name^f^	Sanitation facility construction costs ($USD)	Average connection fees to piped sewerage ($USD)	Public sewer connectivity rate^a^	Informal settlement name^g^	Sanitation facility construction costs ($USD)	Average Connection fees to piped sewerage ($USD)
Kampala	2-stance block: 357	58	100%	Kalimali	NC	NC
Lagos	NC	NC	–	Makoko	NC	NC
Maputo	470	35	100%	Nhlamankulu D	NC	NC
Mzuzu	NC	NC	–	Zolozolo West Ward	NC	NC
Nairobi	104^b^	47	90%	Kosovo Village in Mathare Valley	NC	NC
Bengaluru	543	9	90–95%	Koramangala	543	0^c^
Colombo	548	32	98%	Borella South	645	58
Dhaka	593	296	100%	Kallyanpur Pora Basti	NC	NC
Karachi	282	188	60%	Ghaziabad Sector 11 ½, Orangi Town	122	28
Mumbai	171	124	100%	Siddarth Nagar	NC	NC
Caracas^d^	64	0^c^	–	Terrazas del Alba	64	0^e^
Cochabamba	294	252	100%	San Miguel Km4	252	252
Rio de Janeiro	612	121	100%	Rocinha	NC	NC
São Paulo	639	109	97%	Jardim São Remo	156	63
Santiago de Cali	224	90	87%	Comuna 20	181	86

Note: All costs reported in U.S. dollars. Currency figures were converted to U.S. dollars using market exchange rates (with the exception of Caracas) at the time of data collection. Figures for connection fees represent average costs. Connection fees may vary across the city depending on distance and pipe size. Only Cochabamba, Cali, Colombo, and Maputo had reliable data on connectivity rates. The remaining figures are based on key informant estimates. NC stands for “not connected.”

a Public sewer connectivity rate defined as the proportion of households connected to the sewage service compared to those offered service within the municipal boundaries. For example, if 100 households are offered water sewage service, 50 households are connected, then the household connection rate is 50%.

b This is the cost for a facility within a building already connected to public sewerage.

c There is a wastewater tariff at 15 to 25 percent of the monthly water bill, but the water and sanitation utility has given free connections to this slum without installing water meters. It is unclear if the utility will return to install meters in the future, as water service is highly unreliable without adequate pressure.

d Costs from Caracas were converted using the black-market exchange rate during the time of data collection: July 2017 (Bs8,600 to 1 USD).

e Connection fee to city sewerage is supposed to be charged as a monthly tariff by the water utility, but the utility has not included this yet.

f **Caracas:** INE (Instituto Nacional de Estadística) - INE - Venezuela, 2011a. Censo 2011. http://www.ine.gob.ve/index.php?option=com_content&view=category&id=95&Itemid=26. **Cochabamba:** Interview: Environmental Regulator, National Water Authority, March 2017. **Rio de Janeiro:** Interview: Manager, CEDAE - STS Alegria; Manager, Rio Aguas Foundation, July 2017; IBGE (Instituto Brasileiro de Geografia e Estatística), 2010. Census 2010 (Online Database). http://censo2010.ibge.gov.br/. **São Paulo:** Interview: Specialist in São Paulo household construction, July 2017; Whately and Diniz, 2009. Água e Esgoto na Grande São Paulo: Situação Atual, Nova Lei de Saneamento e Programas Governamentais Propostos. Instituto Socioambiental, São Paulo. **Santiago de Cali:**Emcali (2017). Emcali. www.emcali.com. **Bengaluru:**BWSSB (Bangalore Water Supply and Sewerage Board), 2011. National Census 2011 – Household Amenities table 14. BWSSB. **Mumbai:** Interview: Officer, Sanitation Department, MCGM; Directorate of Census Operations, 2011. District Census Handbook: Mumbai. Series-28. Census of India, Mumbai. **Colombo:** Interview: Engineer, Colombo Municipal Council. Juyl 2017. **Karachi:** Interview: Staff representative, Orangi Pilot Project; Community leaders of Orangi and Baldia; Director, Urban Resource Center, July 2017; **Dhaka:** Interviews: Consultant, World Bank; Representative, WSUP-Dhaka, July 2017; WSUP, 2015. Financial Analysis and Business Model Development on FSM Service. WSUP and UNICEF., WSUP-World Bank, 2014. Base line Survey: Bangladesh LIC WASH Programme, 2014. World Bank. **Kampala:** Interview: Representatives, NWSC; Representatives, KCCA; Representative, ACTogether; Representative, CIDI, July 2017. **Lagos:** Interview: Manager, Lagos State Waste Water Office, July 2017; LBS (Lagos Bureau of Statistics), 2013. Household Survey 2013 Main Report. Ministry of Economic Planning and Budget, Lagos State Government. **Maputo:** Interview: Water and Sanitation Specialist, World Bank; Sanitation Manager, Municipality Drainage Office, July 2017; Water and Sanitation Program Municipality of Maputo, 2014. Caracterização do Saneamento em Maputo, Maputo. **Mzuzu:** Interview: Officer, National Statistics Office, July 2017; National Statistical Office (2010). Population and Housing Census 2008, Spatial Distribution and Urbanisation. National Statistical Office, Zomba. **Nairobi:**KNBS (Kenya National Bureau of Statistics), 2012. 2009 Kenya Population and Housing Census. Analytical Report on Urbanization. KNBS, Nairobi.

g **Caracas:** INE (Instituto Nacional de Estadística) - INE - Venezuela, 2011b. Informe Geoambiental 2011 Distrito Capital. http://www.ine.gob.ve/index.php?option=com_content&view=category&id=68&Itemid=49#.; Interviews: Community leaders, Terrazas del Alba, July 2017. **Cochabamba::** Interview: Regulator, National Water Authority; Administrator, Drinking Water Association, San Miguel Km4, March 2017. **Rio de Janeiro:** Interview: Manager, CEDAE - STS Alegria; Interviews: Director, Rocinha Health Office, July 2017. **São Paulo:**IBGE, 2010. São Paulo (Online Database). IGBE. https://cidades.ibge.gov.br/brasil/sp/sao-paulo/panorama. **Santiago de Cali:**Municipality of Santiago de Cali, 2017. Cali en Cifras (Online Database). http://www.cali.gov.co/planeacion/publicaciones/137802/cali-en-cifras/. Interview: Community leader, Comuna 20, July 2017. **Bengaluru:**BWSSB (Bangalore Water Supply and Sewerage Board), 2011. National Census 2011 – Household Amenities table 14. BWSSB.; Interviews: Community leader, Koramangala, July 2017. **Mumbai:** Interviews: Community leaders, Siddarth Nagar; July 2017.**Colombo:**Department of Census and Statistics, 2017. Census Of Population and Housing 2011. Government of Sri Lanka. http://www.statistics.gov.lk/PopHouSat/CPH2011/index.php. **Karachi:** Interviews: Community leaders, Ghaziabad, July 2017. **Dhaka:** Interviews: Community leaders, Kallyanpur Pora Basti; Representative, NDBUS (Slum Committees), Sep 2017; PMID (Participatory Management Initiative for Development), 2013. Analysis of factors affecting the usage, operation and maintenance of community latrines in low income communities (lics), in Dhaka city, for WSUP Bangladesh LIC WASH Programme 2013–15. **Kampala:** Interview: Representatives, KCCA and ACTogether; Community leaders, Kalimali, July 2017. **Lagos:** Interview: Makoko Representative, Nigeria Red Cross Society; Representative, Lagos State Waste Water Office, July 2017. **Maputo:** Interview: Water and Sanitation Specialist, World Bank; Sanitation manager, Municipality Drainage Office, July 2017. **Mzuzu:** Interviews: Community leaders, Zolozolo West Ward, July 2017. **Nairobi:** Interviews: Community leaders, Kosovo Village, May 2017.

We augmented the city-level data with fieldwork in one informal settlement or low-income neighborhood (in the Latin American urban context) in each city. We used this “case within the case” approach for two reasons: (1) city-level data are usually presented in averages and thus tend to mask extremes at both ends of the socioeconomic distribution, and we were particularly interested in the challenges faced by low-income households; and (2) in many cities, informal settlements are excluded from formal city-level statistics because their land occupation is considered illegal (Jenkins et al., 2014; Sinharoy et al., 2019). To select the informal settlement in each city, the researchers identified a centrally located, well-established settlement that did not represent either the city's “best” or “worst” conditions, but instead represented challenges to water and sanitation access common in similar settlements in the city.

In addition to the city-level and informal settlement data, each researcher wrote a narrative describing the city's land-use patterns, residents' access to sanitation, the rationale for selecting the informal settlement, a description of the institutional landscape of sanitation practices and service provision, and an overview of unique contextual factors. Key insights from reading these qualitative narratives helped us understand unique local circumstances and contextual issues that were important for interpreting the city-level quantitative data. The qualitative data combined with researchers' field observations also provide a way to triangulate the reliability of city-level data obtained from official sources. The qualitative and quantitative data are the basis for the description of the sanitation patterns across the three geographic regions.

Our data address some of the limitations of the JMP data discussed earlier, but also suffer from some of the same challenges. First, some information was collected from households that have a limited understanding of what happens to their waste underground and downstream. Second, there is limited reliable, systematic data about households’ construction of on-site sanitation systems. Approximately 44 percent of residents in the 15 cities live in informal settlements, where much of the sanitation infrastructure is constructed without documenting construction materials, building specifications, and maintenance practices. Table 1 provides an overview of these 15 cities and 15 informal settlements that form the basis of our data.

## 4 Findings and discussion

The discussion of the findings is organized into five parts: (1) urban sanitation patterns across geographic regions, (2) description of what sanitation facilities households use (for example, toilets, latrines, and receptacles), (3) how households manage their human waste, (4) what happens to human waste, and (5) what households pay for sanitation.

### 4.1 Sanitation patterns across geographic regions

We look comparatively at urban sanitation issues across the five cities in each geographic region to highlight regional trends prior to the more detailed discussion (see Table 2, Table 3, Table 4).

#### 4.1.1 Sub-Saharan Africa

Across the five cities in Africa, approximately 8 percent of households have access to a sewer connection, and 92 percent of households use non-sewer methods to dispose of human waste. In these cities there are deficiencies in household-level sanitation services and infrastructure as well as in city-level sanitation systems and strategies. Many households in informal settlements and in the urban periphery have limited access to toilets in their homes. This is a result of the low household incomes as well as the relatively high number of renters. The dominant forms of sanitation in these situations are pit latrines or toilets connected to septic tanks on the plot. Population densities mean that containers fill rapidly, and fecal sludge needs to be removed frequently. The containment, emptying, movement, and associated treatment of human waste is weakly regulated by the public sector, resulting in health risks.

High population densities increase the risks associated with inadequate sanitation. In many inner-city areas where population densities are high, when the pit latrine is full, it is impossible to find an alternative location within the plot to construct a new pit latrine, and thus the pit latrine must be emptied. The relatively high cost of emptying pit latrines means that some households empty pits informally^2^ by allowing the waste to flood out of the latrine during periods of high rainfall (Arsénio et al., 2018). Even when the landlord does pay, regulatory controls are weak, resulting in illegal dumping of fecal sludge. This situation in Lagos illustrates some of the deficits in services. Here, in the absence of a sewerage system, the most common sanitation arrangement used in the city (61 percent of houses) is septic tanks. Legislation in 2017 outlawed the use of pit latrines, VIP toilets, and earth closets. However, in many informal settlements, pit latrines (old and improved types) remain in use, especially in multi-occupancy buildings. On-site fecal sludge is meant to be transported to fecal collection points, but there are only six in the city with an estimated population of at least 23 million (LBS, 2013).

There are three systemic weaknesses that together result in inadequate management of human waste. Pits are allowed to flood out during the rainy season. When fecal sludge is collected, regulation related to safe treatment and disposal is weak. Finally, treatment plants are nonexistent, are not operating, or do not have the needed capacity—thus when human waste reaches the facility, it is not treated. The Nairobi Integrated Urban Development Master Plan, for example, indicates that: “the volume of sewerage collected and conveyed to the STWs accounts for 35 percent of the sewerage generation and the remaining 65 percent are discharged to the environment without treatment” (NCC, 2014). In another example, it is reported in Maputo that the quality of post-treatment discharge from the treatment plant is scarcely better than pre-treatment (Dimene, 2018).

In the African cities, poor regulation is exacerbated by overlapping responsibilities between the multiple agencies involved in the delivery of water and sanitation services to consumers. For example, in Mzuzu sanitation is the responsibility of the City Council. However, the Water Works Act means that they are dependent on the Northern Region Water Board to remove wastewater, but the Water Board is not doing this work. The Mzuzu City Council lacks the funding for legal enforcement, and there is no investment in a sewage management system. Overall, in the five African cities, there were limited efforts to improve the sanitation beyond those focused on privatized individual solutions. In this context, innovation is related to initiatives where households pay to have fecal sludge removed from their dwelling.^3^ Such efforts make a valuable contribution but are not likely to address the need for city-wide sanitation to ensure public health and safety.

#### 4.1.2 South Asia

Across the five cities in South Asia, approximately 53 percent of households have access to a sewer connection, and 47 percent of households use non-sewer methods to dispose of human waste. Of the households that lack a sewer connection, private and communal septic tanks are the most common sanitation solution. However, many of these septic tanks are not constructed properly, cannot be emptied, or release untreated human waste into local drains and waterways. For example, in Dhaka it is estimated that regardless of its containment method, 99 to 100 percent of human waste ends up in drains and nearby rivers untreated (Zaman, 2018). It should also be noted that none of the five cities in South Asia had reliable public fecal sludge collection, transportation, and treatment systems, and none of the five cities treated the wastewater collected through their sewer system.

Bengaluru and Karachi reported a high percentage of households having access to sewers, between 70 and 80 percent. However, Bengaluru's water utility provides water intermittently, and Karachi's sewage system is extremely degraded. In Colombo almost 40 percent of the households in the city have access to sewers, and most of the remaining households rely on private septic tanks. Dhaka and Mumbai report the lowest percentage of households connected to sewers. Dhaka's population largely relies on septic tanks, where households in Mumbai use a mix of on-site solutions, including septic, pit latrines, and self-provisioned pipes that empty wastewater into nearby waterways.

In informal settlements in three cities, Dhaka, Karachi, and Mumbai, there were no household sewer connections. Open defecation was reported in four out of the five cities, including Bengaluru, Colombo, Karachi, and Mumbai. The highest percentage of households without access to sanitation facilities (a private or public bathroom) was found in the informal settlement in Mumbai, which also reported the highest rate of open defecation.

#### 4.1.3 Latin America

Across the five cities in Latin America, approximately 81 percent of households have access to a sewer connection, and 19 percent of households use non-sewer methods to dispose of human waste. The rate of sewer connection to homes was the highest in the Latin American cities; for example, rates in Santiago de Cali were 99 percent, 97 percent in Caracas, and 87 percent in São Paulo. One of the surprises was the number of informal settlements in Latin American cities with access to some of the highest JMP standards of service delivery. For example, in Comuna 20 (in Santiago de Cali, Colombia) drinkable water is available 24 hours a day 7 days per week and 99 percent of residents had a toilet in their home. But the majority of Comuna 20's population obtains water through illegal connections and residents use rainwater drainage pipes to dispose of household sewage, thus contaminating the local rivers.

In Caracas, most of the population have connections in their homes to piped water and sewers. But generally, informal settlements have intermittent water service and formal areas have continuous service. In the informal settlement of Terrazas del Alba in Caracas there are 13 publicly built housing projects with formal apartments. The main challenge is the lack of reliable water service that then creates problems with the sewer system. Also, when there are obstructions, sewage flows into the street. This example has wider relevance because 100 percent of households are connected to piped water, but water service is intermittent, and contributes to sewer blockages, and this results in the whole system not functioning as intended.

In summary, across the three geographic regions the proliferation of informal or low-income settlements created challenges to providing universal access to sanitation services. Sometimes the geographic or physical characteristics of these settlements made sanitation provision more difficult; for example, settlements that are built into steep hillsides. There are a number of physical characteristics that make urban sanitation both challenging and an urgent public concern. First, there are those cities like Dhaka, Kampala, Lagos, and Mzuzu where the water table is high, and the absence of a well-maintained sewer system to convey human waste away from the plot or a properly functioning and maintained on-site sanitation system has the potential to contaminate ground water supplies.

Given that most of our 15 cities, in one form or another, rely on water to convey human waste away from populated areas, water shortages pose a serious public health risk. A number of cities, namely Cochabamba, Maputo, Mzuzu, Nairobi, Rio de Janeiro, and São Paulo, have experienced severe water shortages in the recent past resulting from poor water management, inappropriate land use patterns, and climate change, thus making it more difficult for sanitation systems to work as intended. In Kampala, Rio de Janeiro, Caracas, and Nairobi, water recharge zones have been covered with impervious surfaces, and access to natural waterways has become increasingly difficult. This has made the use of waterways for conveying human waste away from residential areas increasingly difficult. Equally challenging are those cities that regularly experience flooding, such as Lagos, where the absence of a piped sewer system combined with the use of on-site sanitation increase the risk of human contamination.

### 4.2 What sanitation facilities do households use?

Access to sanitation starts with having a place—a sanitation facility— in which to defecate and dispose of human waste (see Table 2). Private sanitation facilities are located inside a house or on the house plot and are not shared. Shared sanitation facilities are privately managed and shared by more than one household. Communal or public sanitation facilities are managed by a range of actors, including communities, nongovernmental organizations (NGOs), and local governments. Households categorized as having no sanitation facilities dispose of their fecal matter in open spaces or engage in other forms of open defecation.

As seen in Table 2, the highest percentage of households with a private sanitation facility was found in Santiago de Cali, Caracas, Cochabamba, and São Paulo, at 99 to 100 percent. The lowest percentages, less than a third, were found in Kampala, Lagos, and Nairobi. For shared toilets, the highest percentages were 72 percent in Lagos, 60 percent in Kampala, and 51 percent in Mzuzu, all cities in which many residents rent space in compounds that have shared facilities.

Of the 15 informal settlements, the percentage of households with a private sanitation facility was 100 percent in four settlements: Caracas, Cochabamba, Karachi, and São Paulo.^4^ This figure was 99 percent in settlements in Santiago de Cali, 97 percent in Rio de Janeiro, and 93 percent in Colombo. The percentage with shared sanitation was highest in our selected informal settlements in Nairobi (85 percent) and Lagos (65 percent).

In the informal settlements, the percentage of households without access to facilities was highest in Mumbai, at 55 percent, and 10 to 15 percent in informal settlements in Mzuzu and Lagos, respectively. This is consistent with our findings that cities in South Asia and sub-Saharan Africa had the highest rates of open defecation. Sanitation service provision and use in informal settlements differs considerably based on a variety of factors, including when the neighborhood was established, landownership, residential density, the location of the settlement, the availability of land, the extent to which standards and regulations are enforced, and collective practices (Leong, 2020).

### 4.3 How do households manage their human waste?

Within cities and even within informal settlements, households manage human waste in different ways, which include the use of sewers, private or communal septic tanks, various types of pit latrines, composting toilets, buckets, hanging toilets, smaller forms of containment, open defecation, and self-provisioned drains (see Table 3). Although septic tanks and pit latrines can hold waste for longer periods of time, they need to be carefully emptied. On the other hand, self-provisioned drains, hanging toilets, and other informal types of containment usually result in untreated waste being frequently disposed of directly into the local environment. In Maputo 80 per cent of the population lives in a peri-urban area or “*caniço* city” (*cidade de caniço*) where there are no formal sanitation services (Dimene, 2018). Across the city, 90 percent rely on septic tanks and pit latrines. Table 3 describes households’ access to these various disposal methods at the city level and in one informal settlement (Dimene, 2018).

As is true of other categories, household access to sewers ranges widely in all three regions. Toilets connected to sewers need regular water supplies for flushing. Only Colombo, São Paulo, and Santiago de Cali have public-provided water available continuously, 24 hours a day, seven days a week. Bengaluru reports that 79 percent of households use sewers, but water is only available on average for 3 h, three days a week across different locations in the city. Cities in sub-Saharan Africa had the lowest percentage of households with access to sewers, ranging from between 0 percent in Lagos and Mzuzu to 10 percent in Kampala. In the event of intermittent water supply, sewers will not function properly and there will be an increased risk of contaminating the piped water supply.

Private septic tanks are reportedly a main alternative for many cities with lower rates of household sewer connection. They serve 75 percent of households in Dhaka, 61 percent in Lagos, 59 percent in Colombo, and 49 percent in Maputo. However, it is important to qualify that many of these receptacles are not properly constructed, and thus do not function as intended. For example, key informants from Dhaka reported that more than 80 percent of these “septic tanks” do not have soak pits and directly connect to drainage. This is consistent with a 2016 study that reported that half of Dhaka's households use a “sealed box [that] discharges to the drainage system” (Furlong, 2016; Ross et al., 2016).

In sub-Saharan African cities, low construction costs mean that pit latrines are an important mechanism for immediately disposing of human waste in the absence of alternatives; for example, in Mzuzu and Kampala, 84 percent and 60 percent of households use pit latrines, respectively. Several field interviews also highlighted the low quality of pit latrine construction, emptying practices, and schedules, and, as a result, their overall inability to prevent contamination. Risks may be especially high where households rely on nearby groundwater sources for domestic water use, especially from shallow and unprotected wells; and where high residential densities mean that pits fill up rapidly and need to be emptied.

In cities in the global South, households’ untreated human waste is often disposed of using a pipe that empties into a nearby waterway or storm water drainage channel. Enumerators in Bengaluru, Caracas, Cochabamba, Colombo, Kampala, Karachi, Lagos, Nairobi, and Santiago de Cali all acknowledge this method of disposal exists in their cities, but there is no reliable way to estimate the percentage of households that use this method. In Dhaka this method is primarily used in the urban periphery. In some cities this method functions like an open sewer and is likely included in our sewer estimates, thus leading to an overestimation of how many households have sewer access.

Composting, bucket, and hanging toilets are used by 5 percent of households in Kampala and 1 percent of households in Lagos and in Bengaluru. Open defecation is practiced in cities in all three regions, but it is most common in Karachi (15 percent) and Mumbai (10 percent).

In the informal settlements where field research was conducted, nine out of 15 settlements—including those in Dhaka, Karachi, Mumbai, Rio de Janeiro, and in all the sub-Saharan African cities—did not have access to sewer infrastructure. There is also a discrepancy between the number of informal settlements that reported that 90 to 100 percent of households are connected to sewers, such as those in Bengaluru, Caracas, Cochabamba, and Santiago de Cali, because sewers need constant water supplies to function properly, yet only the settlement in Santiago de Cali has access to continuous piped water. Based on field observations in African cities, problems accessing sanitation are particularly acute for renters both because landlords commonly shut off access to waterborne sanitation when water is not available, and because pit latrines may not be maintained.

Households in the informal settlements we examined also use private septic tanks, with the highest rates found in Colombo (49 percent) and Maputo (44 percent). Similar to the city-level findings, in four out of the five sub-Saharan African informal settlements, households rely on pit latrines. Self-provisioned drains are also widely used in the informal settlements in Karachi, Mumbai, Nairobi, Rio de Janeiro, and São Paulo. Open defecation was only found in the two informal settlements where we conducted field research, in Siddarth Nagar in Mumbai (55 percent) and in Rocinha in Rio de Janeiro (6 percent).

### 4.4 What happens to human waste?

In geographic terms, patterns of access to sewers and fecal sludge treatment follow national-level data for urban areas, with high percentages in Latin America, lower percentages in South Asia, and the lowest percentages in sub-Saharan Africa. Two cities in sub-Saharan Africa, Lagos and Mzuzu, have no public sewers. Another finding is that although many sewage systems collect human waste, as already noted there is wide variation in how much of it is treated and the level to which it is treated. For example, in Cochabamba, 80 percent of human waste is collected by a sewer system, but only 48 percent is delivered to a treatment plant (the remaining 32 percent is discharged to open surface water bodies). An extreme situation is found in Caracas, where 97 percent of human waste is conveyed via sewer and 0 percent is sent to a treatment plant. There is also a discrepancy between how much human waste is sent to the treatment plant and how much is actually treated because treatment plants are overwhelmed or not functioning properly.

The amount of on-site sanitation that is not emptied (“no removal”) seems low given qualitative information. This might be because “flooding out” pit latrines (an informal coping mechanism) is against sanitation regulations and therefore underreported and not captured by surveys. In contrast, there are other cities where a large number of households use on-site sanitation and the percentage of human waste that is safely managed is higher than the percentage of households connected to sewers. For example, in Mumbai, 28 percent of households are connected to sewers, yet 56 percent of human waste is safely managed; in Kampala, only 10 percent of households are connected to sewers, yet 29 percent of human waste is safely managed; in Lagos, 0 percent of households are connected to sewers, and 45 percent of human waste is safely managed. This underscores the important work of local authorities in regulating the safe management and treatment of fecal sludge. However, we remain cautious about just how safely this fecal waste is being managed because of findings related to the lack of adequate treatment of waste within waste treatment plants.

**Fig. 1:**
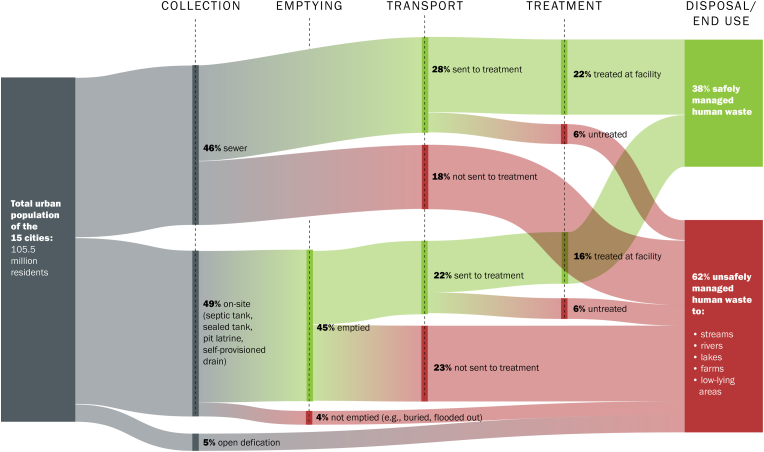
In 15 global South cities, 62 percent of human waste is unsafely managed.

Fig. 1 illustrates in 15 cities studied, when weighted by population in each city, the majority of human waste ends up largely untreated in local water ways. However, the means of conveying human waste to these waterways differs. Sometimes human waste is contained on-site for a period of time; other times it is conveyed away from the plot through either a self-provisioned drain or more formal public sewage systems. In the 15 cities if any human waste was treated before disposal, the proportion was relatively modest. In many of the 15 cities there was no functioning wastewater treatment plant.

### 4.5 What do households pay for sanitation?

The cost of sanitation is closely related to the question of access.^5^ When households do not have access to a public sewer connection, they often pay to provide their own on-site sanitation solution. Table 4 shows the cost of constructing and emptying various on-site sanitation options in the 15 cities and respective informal settlements.

In summary, there is wide variation in costs incurred by households. In the case of septic tanks, some of the differences are explained by differences in construction, the size of the tank, the quality of materials used, and the condition of the ground where the tank is installed.^6^ The average cost of building a household septic tank in Karachi is approximately $165; compare this to Nairobi, where a conventional concrete tank costs around $1,987, but a smaller plastic “biodigester” tank is $662. In Dhaka, communal tanks are widespread and the average communal septic tank ($711) serves 5 to 20 families. The cost of emptying a private septic tank (per tank, per time) also varies considerably, between $24 and $29 in Colombo and Bengaluru, respectively, to $313 in São Paulo and $565 in Karachi.

In general, the cost of building a septic tank in the informal settlements was the same or lower compared to the average cost at the city level, except in the cities of Colombo and Cochabamba, where it was more expensive. Although the cost of constructing septic tanks is relatively inexpensive in Latin America, the cost of emptying them is relatively high compared to the costs in other regions. It is important to keep in mind that these costs are averages and are not based on a single standard-sized tank.

The cost of installing a pit latrine also varies for a range of reasons. For example, a pit latrine might consist of a shallow hole surrounded by four posts with material draped around them for privacy. Or it might be surrounded by a more substantial building that has a lockable door, a slab foundation, and a ventilation pipe. Other factors that affect cost are the materials and construction needed to fit the location's topography, water table, and geology. The most basic pit latrines with no slab are relatively inexpensive to build if the land is available; for example, $16 in Maputo, $23 in Mumbai, and $24 in Colombo and Dhaka.^7^

Pit latrines are also relatively inexpensive to construct in informal settlements. In Dhaka's informal settlement, they cost $36 to construct; in Maputo's settlement, it costs $16 to construct a basic pit latrine with no cement slab. Pit latrines with slabs and VIPs are more expensive, respectively two to four times and three to six times the price of a basic pit latrine.^8^ The cost of emptying pit latrines ranges between $6 for manual evacuation in Dhaka to $96 for mechanized pump out in Kampala. It should be noted that many landlords and their tenants in informal settlements refuse or cannot afford to pay for these services. Consequently, pits are partially emptied or “flooded out” during the rainy season. In Mzuzu, when a pit latrine is full, it is closed and a new pit is dug; this is only possible in low-density areas.

All cities in the study except for Lagos and Mzuzu have public sewer systems that served some proportion of the urban population. For those with sewer connections, there is the initial cost of connection and then the costs of constructing the latrine. In some cases, this is incorporated into the dwelling; in other cases it is a stand-alone unit outside of the house. There are ongoing charges for water (whether formally or informally supplied) and for wastewater treatment (if it is a formal connection).

Our on-site sanitation cost data in Table 4 shows the wide range of sanitation options and the varying costs for the household. Table 5 describes the costs associated with building a latrine in a household (including labor and materials, and excluding land costs) and connecting to the municipal sewer system. From the household perspective, on-site services are not necessarily less expensive than off-site services, and in many cases on-site services are more expensive.

The cost of building a sanitation facility (toilet) ranges from as low as $64 in Caracas to as high as $639 in São Paulo. Drawing on data from 12 cities, the cost per household for a sewer connection ranges from $9 in Bengaluru to $296 in Dhaka. The cost of connecting to the public sewer system—where available—is substantially lower in sub-Saharan Africa, ranging from $35 in Maputo to $58 in Kampala. Seven of the 15 informal settlements are at least partially connected to a public sewer system. The cost of constructing a household sanitation facility ranges from $64 in the informal settlement in Caracas to $645 in Colombo's informal settlement, which is comparable to citywide costs.

Sanitation often involves a relatively large one-time capital investment, unless people are buying services on a pay-per-use basis (or through a subscription to a local pay-per-use facility). For those able to access sewers, the 15-city study shows that the one-time connection and construction costs for households in informal settlements can exceed 300 percent of their average monthly household income. For those households using on-site sanitation (pit latrines and private septic tanks), the one-time construction, which is usually borne entirely by the home owner, can exceed 700 percent of average monthly incomes in informal settlements. If sewers are available, they are likely to be the most affordable option for households to achieve safe and reliable sanitation.

In some cases, toilets are not connected to sewers but rather to informally constructed, self-provisioned pipes that remove the human waste from the immediate vicinity. Researchers in nine of the 15 cities—Bengaluru, Caracas, Cochabamba, Colombo, Kampala, Karachi, Lagos, Nairobi, and Santiago de Cali—acknowledge the widespread use of self-provisioned drains. This is clearly unsafe, and is also indicative of households’ inability to afford formal connections (although these may not be available in informal settlements).

The high cost of water is relevant to urban sanitation because sewers require continuous water supplies to work properly. Additional affordability challenges for Africa – beyond the cost of paying for formally supplied piped water – are illustrated in the elaboration from Kampala:

“Most respondents cannot afford the cost of a private water connection (about USh 250,000) and even then, income characteristics, tenure considerations, and NWSC billing procedures preclude such possibilities in informal settlements. Hence the dominant service level for water supply is typically public standpipes or pre-paid meter systems, where small quantities are purchased several times a day per family. But the water price at standpipes is five times the NWSC domestic tariff.” (Lwasa, 2018)

The cost of water was most affordable in the South Asian cities, compared to the cost of water in the Latin American and sub-Saharan African cities.

The UN Water Supply and Sanitation Collaborative Council suggests that to be affordable, a household's monthly combined expenditures on water and sanitation services should not exceed 5 percent of its income (UN, 2010). In our analysis of the 15 cities, we found that in informal settlements in four cities, buying minimum WHO recommended quantities of piped water (50 L per person per day in a non-emergency situation) from the water utility (which is usually the least expensive source of water) exceeds 3 percent of income (Beard and Mitlin, 2021). This is an underestimation of the true cost paid because many households in informal settlements cannot fulfill all their water needs with piped water, and thus spend a larger share of their income on water purchased from vendors and alternative sources (Beard and Mitlin, 2021).

Affordability is typically measured as a percentage of income. Looking at incomes in the informal settlements, Table 1 suggests that water- and sanitation-related expenditures of 5 percent of monthly household income should be between $2.40 and $25 a month in Africa and Asia. The cost to empty a septic tank and pit latrine exceeds $25 in almost all cities, just one illustration of the affordability challenge. Research suggests that low-income households in urban sub-Saharan Africa can only afford to pay between $3 and $4 a month for sanitation.^9^ They may be unable to pay higher rent for a room with sanitation facilities, and they may lack access to pay toilets. Tenants are dependent on their landlords and the availability of public sewers. Sanitation investments are often associated with higher rent, exacerbating problems of affordability. Providing safe, reliable, and affordable sanitation for all will require massive public investment and continued subsidies.

What do we find from our study with respect to affordability? First, that the percentage of income spent is an insufficient measure. The absolute amount spent also has to be considered to know if sanitation is affordable. Second, when those absolute amounts are considered it is evident that lack of income is a major constraint to accessing adequate sanitation. Third, that household costs for sewer connections were on par or less expensive than building a private septic tank (Table 4, Table 5). Although sewers represent a significant investment on the part of cities, sewers eliminate the need for households to pay to build a septic tank, pay to empty it, and pay to transport and treat fecal sludge, although households may incur a monthly sanitation fee for sewer services. In all cities, the costs associated with on-site sanitation systems or connecting to sewers (where they exist) are high for low-income groups in proportion to monthly household income. Sewers ensure that part of the investment costs are paid by the public agencies through taxation and long-term borrowing, enabling repayments to be spread over time. Without sewers, households have to cover the costs themselves.

## 5 Conclusion

There is a significant underestimation of the magnitude of the urban sanitation crisis in the global South. The JMP sanitation categories should be revised to provide a more accurate and useful picture of sanitation practices, access, and risks in cities. In addition, city and sub-city level data are needed regarding provision for sanitation and who is responsible for what parts of the sanitation service chain, as well as data on the availability and affordability of different sanitation services and practices from the perspective of the user. Criteria used to assess public health and safety must take the urban built environment and residential density into account.

With the exception of some cities in Latin America, most cities in the global South currently have a combination of off-site and on-site sanitation systems. Off-site solutions place the least burden on a household in terms of cost and risk; however, they require the largest initial capital investment as well as a strong capacity for municipal planning, governance, and financial management on the part of local governments and sanitation utilities. Our data shows a serious deficit in the ability of local governments and utilities to treat the waste that is being collected through sewer systems. This needs to be addressed.

Most on-site solutions require less capital investment on the part of the city or national government. On-site sanitation solutions place enormous responsibility on individuals, households, and communities. In dense urban settlements, safe on-site solutions require human waste to be stored, moved, and treated and these processes require considerable state capability to manage, regulate, and enforce safe practices. Some policymakers will argue that governments can regulate on-site sanitation solutions and that on-site approaches will create opportunities for the private sector. However, on-site sanitation solutions require far more capacity to regulate on the part of local government and sanitation utilities—capacity that does not currently exist in most cities. On-site solutions also require significant ongoing household expenditures that does not appear to be affordable.

The patterns of urban informality, sanitation conditions, and practices are very different in the three regions. In many Latin American cities services have been extended to informal settlements. For example, Santiago de Cali has high sanitation coverage in its informal settlements. In Caracas there is also high coverage with conventional piped water and sewer networks, but informal settlements receive intermittent water service and poor-quality water. In the informal settlements in South Asia, services have not kept pace with population growth, and in some cases services are not provided to prevent future land tenure claims. In the African cities, there is very limited investment capacity for developing public sanitation systems. In some cities, even the middle- and high-income dwellers are not served. For example, in Maputo, middle- and high-income households share the same challenges as informal settlements.

In summary there is an urgent need to address the sanitation crisis in cities in the global South. This crisis poses particular public health risks in high density urban areas because of the inability of on-site systems to ensure safe sanitation practices for the entire sanitation service chain. In the absence of universal access to safe, reliable, and affordable sanitation services households continue to provide their own solutions, resulting in practices that are costly to household, risky to public health, and damaging to the natural environment. Sanitation access for all will require a sustained political commitment to providing a publicly coordinated sanitation system regardless of a household's land tenure status or ability to pay.

## Funding

The research was funded by: 10.13039/100000865Bill and Melinda Gates Foundation (grant number OPP1175744), United Kingdom Department for International Development, Stephen M. Ross Philanthropies Denmark Ministry of Foreign Affairs, Ireland Department of Foreign Affairs and Trade, Netherlands Ministry of Foreign Affairs, Swedish International Development Cooperation Agency, United Nations Development Programme.

## Author credit statement

**Victoria A. Beard:** Conceptualization, Methodology, Formal analysis, Writing. **David Satterthwaite:** Conceptualization, Methodology, Formal analysis, Writing. **Diana Mitlin:** Conceptualization, Methodology, Formal analysis, Writing. **Jillian Du:** Conceptualization, Methodology, Formal analysis, Writing.

## Declaration of competing interest

The authors declare that they have no known competing financial interests or personal relationships that could have appeared to influence the work reported in this paper.

## Footnotes

1 For more information on how SFDs are calculated, see SuSanA, n.d. Up-Scaling Basic Sanitation for the Urban Poor (UBSUP) in Kenya.

2 In many cases the pathways to the latrines are so narrow that it is impossible for formal emptying services to access the areas.

3 See, for example, Sanergy's provision of household level container-based sanitation and the “gulper” technology that allows fecal sludge to be removed from pit latrines that cannot be accessed by fully mechanized removal systems.

4 It should be noted that the informal settlement in Karachi, Orangi, is an example of an informal settlement that has had active community participation in sanitation for more than 30 years; see Hasan, 2006. Orangi Pilot Project: the expansion of work beyond Orangi and the mapping of informal settlements and infrastructure. Environ. Urban. 18, 451–480. https://doi.org/10.1177/0956247806069626.

5 In this context we refer to on-site sanitation as a cost instead of a price because many households are providing their own sanitation solutions and the price is not solely mediated by the market.

6 We converted all local currencies to dollars using current exchange rates, except in the case of Caracas where we used the “black market” exchange rate.

7 To facilitate ease of interpretation, all costs are calculated using market exchange rates.

8 There are also very rudimentary latrines that cost almost nothing—shallow pits, four posts, and material walls.

9 See Banana et al., 2015. Sharing reflections on inclusive sanitation. Environ. Urban. 27, 19–34. https://doi.org/10.1177/0956247815569702, supported by interviews with Slum/Shack Dwellers International (SDI) community leaders.
